# Personality Traits Related to Binge Drinking: A Systematic Review

**DOI:** 10.3389/fpsyt.2017.00134

**Published:** 2017-07-28

**Authors:** Ana Adan, Diego A. Forero, José Francisco Navarro

**Affiliations:** ^1^Department of Clinical Psychology and Psychobiology, University of Barcelona, Barcelona, Spain; ^2^Institute of Neurosciences, University of Barcelona, Barcelona, Spain; ^3^Laboratory of Neuropsychiatric Genetics, School of Medicine, Universidad Antonio Nariño, Bogotá, Colombia; ^4^Department of Psychobiology, University of Málaga, Málaga, Spain

**Keywords:** binge drinking, heavy episodic drinking, personality traits, impulsivity, sensation seeking, neuroticism, anxiety, prevention

## Abstract

The pattern of alcohol consumption in the form of binge drinking (BD) or heavy episodic drinking has increased notably worldwide in recent years, especially among adolescent and young people, being currently recognized as a global health problem. Although only a minority of binge drinkers will develop a substance use disorder, BD may have negative personal and social consequences in the short and medium term. The objective of this article is to review the findings on personality traits related to binge drinkers and to emphasize the aspects that should be examined in order to make progress in this area. The main characteristics of personality related to the practice of BD, regardless of the theoretical model used, are high Impulsivity and high Sensation seeking, as well as Anxiety sensitivity, Neuroticism (Hopelessness), Extraversion and low Conscientiousness. The data obtained may have theoretical implications to elucidate the endophenotype of BD, but they are especially useful for their preventive applications. Integration into prevention programs of emotional self-control skills, decision-making, social skills, and strategies to manage negative emotions will minimize the risk factors or consequences of BD associated with personality and will improve their effectiveness. In the future, it is necessary to harmonize a common measurement instrument for the assessment of personality, develop longitudinal studies with large samples that also integrate biological and neurocognitive measurements, and determine the reciprocal relationship between personality and BD together with its modulating variables, as well as the possible cultural differences.

## Introduction

The pattern of binge drinking (BD) or heavy episodic drinking is increasing and expanding worldwide (1). Although it is recommended to define the BD as the consumption of high quantities of alcohol (≥4/5 drinks for women/men) within a time period of 2 h (2), there is no consensus and it is frequent to consider the consumption in one occasion/sitting. BD supposes an important public health problem of which it is still necessary to know better the vulnerability factors responsible for its initiation, maintenance, or increase in frequency and intensity.

Individuals who practice BD are exposed to numerous adverse psychological and health-related outcomes (3). Acute alcohol intoxication includes accidents caused by driving while intoxicated, unwanted sexual behavior, and fights or other disruptive behaviors with possible legal implications. The repeated pattern of alcohol intoxication is related to cognitive impairments (4, 5), worse health-related quality of life (6), and an increased risk of suffering psychiatric symptomatology/disorders (7, 8).

The study of the characteristics or personality traits associated with the engagement of BD patterns, such as possible factors of risk or vulnerability, as well as the influence that consumption has on them, is of great theoretical and applied relevance. In this sense, it is now being suggested that personality is an endophenotype that is sensitive for identifying different subtypes of alcohol use disorders (9), also considering that the modification of behaviors linked to extreme personality traits may be beneficial for prevention and treatment of BD. Focusing on studies in adolescents and young people is not only motivated by the time of appearance and boom of the practice of BD but also because in this period of development and maturation of the organism the biological and behavioral impact of alcohol intoxications is more serious (4, 5).

This article reviews existing data on personality characteristics associated with the practice of BD (considering the several definitions) and its evolution, as well as the possible relationships with other variables that increase the risk or are protective for the maintenance and problematic evolution of the consumption. We also mention limitations and future directions that may allow for progress in this area of research.

## Method

**Figure 1:**
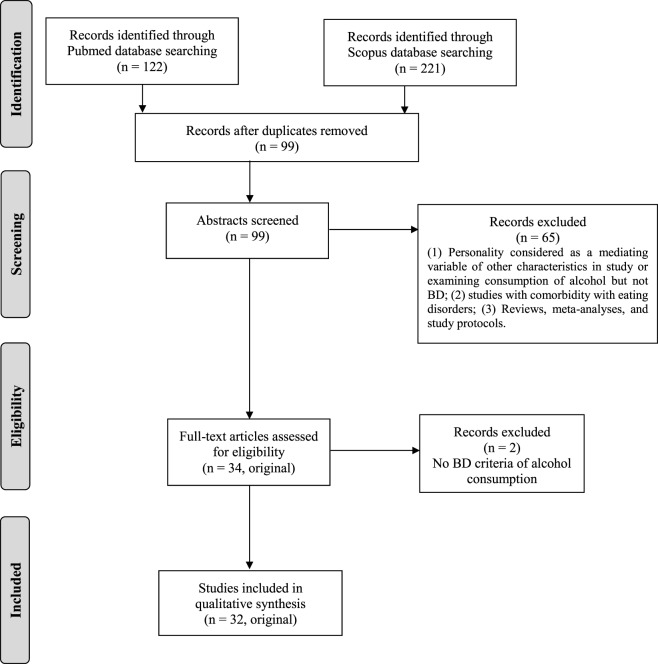
Flowchart for the selection of articles, bibliographic search from January 2006 to February 2017.

The search, selection, and critical assessment of relevant studies were performed according to the PRISMA guidelines (10). The data search was conducted through the computerized databases PubMed and Scopus with “Binge drinking” (or “Heavy drinking” or “Heavy episodic drinking”) and “Personality” as keywords, from January 2006 to February 2017 (Figure 1). The search and selection were performed independently and blindly by two authors, and discrepancies resolved by consensus.

## Results

Table 1 presents the studies included in the present review, considering the sample characteristics, BD criteria, assessment of personality, and main results.

**Table 1:** Results of the empirical studies published on binge drinking (BD) and personality traits (from 2006 to February 2017), according to the characteristics of the sample, BD criteria, and the assessment instruments used.

Reference	Sample characteristics	BD criteria	Personality instruments	Main results
Adan (11)	160 university students, 80 binge/80 non-binge drinkers (40 women in each group) 21.38 years	≥4/5 drinks for women/men within 2 h at least once in the previous 30 days	Functional Impulsivity and Dysfunctional Impulsivity (FIDI)	The scores in Dysfunctional Impulsivity were higher in the BD group compared to the non-binge group, while no differences were found in Functional Impulsivity.Men showed a higher degree of Functional and Dysfunctional Impulsivity, although in Dysfunctional Impulsivity significant differences were observed only in the BD group.Circadian typology controlled (all intermediate type).

Adan et al. (12)	140 university students, 70 binge/70 non-binge drinkers (40 women in each group) 21.33 years	≥4/5 drinks for women/men within 2 h at least once in the previous 30 days	Zuckerman and Kuhlman personality questionnaire (ZKPQ)	Binge drinkers presented higher scores in the Neuroticism-Anxiety and Impulsivity-Sensation seeking dimensions than non-binge drinkers.The Neuroticism-Anxiety results are mainly due to the women’s scores, while the higher scores in Impulsivity-Sensation seeking are due mainly to the men’s scores.Circadian typology controlled (all intermediate type).

Ashenhurst et al. (13)	2,245 students (1,345 women) 18.4 years at inclusion	≥4/5 drinks for women/men at a sitting	Zuckerman and Kuhlman personality questionnaire (ZKPQ): only Impulsive and Sensation seeking scale	**Longitudinal study:** 6 years follow-up, from end of high school to 2 years after college.Impulsivity and Sensation seeking at inclusion were higher in frequent and increasing trajectories compared to moderate, occasional, rare, or decreasing trajectories. Only increasing trajectory enhanced the Impulsivity scores, while the frequent trajectory showed the highest decrease in Impulsivity. Sensation seeking decreased in all groups, but in the increasing trajectory scores were still the highest.

Balodis et al. (14)	428 university students (152 women) 20.00 years.	≥4/5 drinks for women/men on drinking occasions	Barrat Impulsiveness Scale (BIS); Comprehensive Effects of Alcohol Questionnaire (CEOA)	Binge drinkers did not exhibit higher Impulsivity levels than non-binge drinkers.BIS scores correlated with the number of drinks consumed and the length of the BD episode (risk for impaired control).Women reported higher sociability and sexuality expectations, whereas men reported greater tension reduction expectations with alcohol.

Bhochhibhoya et al. (8)	334 university students (166 women) 20.68 years.	≥4/5 drinks for women/men on one occasion the past 30 days	Denollett’s type-D personality scale (DS14)	People with higher risk of type-D personality and mental disturbance (Kessler Psychological Distress Scale) showed a higher probability for drinking more alcohol (including a BD pattern).

Biolcati et al. (15)	721 adolescents (61% girls) 15.98 years.	≥4/5 drinks for women/men in one night	Boredom Proneness Scale (BPS)	Boredom proneness predicted drinking social expectancies. Disinhibition and relief from pain played an important mediating role between Boredom and alcohol outcome.

Bo et al. (16)	162 university students (82 women) 18–25 years.	Binge score based on: number of drinks/h, number of times intoxicated, and percentage of time drunk when going out drinking	UPPS-P Impulsive Behavior Scale	BD was associated with Sensation seeking, but when controlling for sex, age, and global alcohol consumption, only the impulsivity component of Negative urgency predicted severity of BD.

Carlson and Johnson (17)	302 university students (198 women) 20.74 years.	≥4/5 drinks for women/men within 2 h during the past year	Barrat impulsiveness scale (BIS)	Impulsivity scores were related to BD frequency.Impulsivity was not significantly associated with drinking for participants with low levels of positive expectancies but was increasingly related to alcohol use with higher levels of positive expectancies.

Carlson et al. (3)	293 university students (199 women) 20.73 years.	≥4/5 drinks for women/men within 2 h during the past year	Barrat Impulsiveness Scale (BIS); Sensation Seeking Scale (SSS-V); Aggression Questionnaire (AQ)	Controlling for demographic variables (sex, age, residing away from parents, residing off campus, and parental socioeconomic level), only Motor impulsiveness, Thrill and adventure seeking, and Boredom susceptibility were associated with BD frequency.With the exception of verbal aggression, aggressive personality traits were no related to BD.

Castellanos-Ryan et al. (18)	76 adolescents (55 girls) 14.00 years at inclusion.	≥4/5 drinks for women/men on 1 or more occasions in the past 6 months	Substance Use Risk Profile Scale (SURP); Impulsivity and Sensation seeking dimensions	**Longitudinal study:** 2-year follow-up (14–16 years)Sensation seeking was associated with the unique variance in BD frequency and part of this overlap was mediated by reward-related disinhibition (go/no-go task). Impulsivity was associated with a 2-year average of conduct disorders symptoms, and deficits in response inhibition (visual tracking Stop task) partially mediated on it.

Clark et al. (19)	142 binge-drinking university students (98 women) 21.2 years.	≥4/5 drinks for women/men consumed in a row	Neuroticism-Extraversion-Openness (NEO) Five Factor Inventory; Positive Alcohol Metacognitions Scale; Negative Alcohol Metacognitions Scale	Conscientiousness and positive alcohol metacognitions about cognitive self-regulation were the only two predictors of weekly levels of alcohol use when controlling for sex. In males, low Conscientiousness and positive alcohol metacognitions were risk factors for increased levels of alcohol use in BD.

Doumas et al. (20)	346 high school students (177 women) 17.2 years.	≥3/5 drinks for women/men in a row in a2-h period during the last 2 weeks	Zuckerman and Kuhlman personality questionnaire (ZKPQ): Only Impulsive Sensation seeking scale	Higher levels of Impulsive Sensation seeking were associated with higher levels of BD. Protective behaviors in manner of drinking modulated the results, whereas use of more drinking strategies in participants with high Impulsive Sensations seeking diminished the frequency of BD.

Flett et al. (21)	207 university students (131 women); 70 binge drinkers (37 women) 18.89 years.	≥5 drinks in a single occasion in the past 2 weeks	Multidimensional Perfectionism Scale; Frost Multidimensional Perfectionism Scale	Binge drinkers, with two or more binge episodes in the past 2 weeks, had lower levels of self-oriented Perfectionism and higher levels of parental criticism.

Lac and Donaldson (22)	506 university students (351 women) 19.34 years.	≥4/5 drinks for women/men at least once in the past 30 days	Sensation seeking 8-items version; Big Five Personality taxonomy	Binge drinkers presented higher scores in Sensation seeking and Extraversion, and lower in Neuroticism, than non-drinkers and moderate drinkers. Sensation seeking was the strongest personality trait for men and Neuroticism for women, which discriminated the drinking type (non-drinkers, moderate drinkers, and binge drinkers).

Lang et al. (23)	206 university students (110 women) 19.5 years.	≥4/5 drinks for women/men on one occasion in the past month	Brief Sensation Seeking Scale-4 (BSSS-4)	Students with high Sensation seeking reported drinking more and had a higher frequency of BD than students with low levels. They also experienced more positive consequences as a result of their drinking.Positive consequences mediated the relation between Sensation seeking and both drinking and BD frequency.

Lannoy et al. (24)	867 binge drinkers (374 women) and 924 non-binge drinkers (388 women) university students 21.44 years.	>5 drinks for women/men per occasion (alcohol unit 10 g) having between 0.5 and 4 occasion per week	UPPS-P Impulsive Behavior Scale; Drinking Motives Questionnaire-Revised (DMQ-R)	Binge drinkers had lower scores of Negative urgency and Sensation seeking and higher scores on Lack of premeditation than non-binge drinkers.Binge drinkers were not a unitary group. Drinking profiles:(1) Emotional: higher values for Negative urgency and Sensation seeking; (2) recreational: higher Lack of premeditation and Lack of perseverance; and (3) Hazardous: moderate to high values of drinking motives (enhancement, social order, coping, conformity).

Leeman et al. (25)	312 university students (184 women) ≥18 years.	≥4/5 drinks for women/men at a single sitting	Sensation Seeking Scale (SSS, form V), only Disinhibition subscale	BD predictors were sex (male), intensity of best friend’s drinking, and Sensation seeking (disinhibition).

Leeman et al. (26)	3,106 high school students (1,696 girls) 15.86 years.	≥4/5 drinks for women/men in a row the past 30 days	Zuckerman and Kuhlman Personality Questionnaire (ZKPQ), only Impulsive Sensation seeking scale	Impulsivity, Sensation seeking, having a paid part-time job and non-participation in extracurricular activities, were positively associated with BD and more alcohol consumption overall. Only Impulsivity had significant associations with related problems (drug and gambling).

Mackie et al. (27)	393 adolescents (265 girls) 13.00 years at inclusion	≥4/5 drinks for women/men on one occasion in the past 6 months	Substance Use Risk Profile Scale (SURP); Brief Symptom Inventory (BSI) for depression and anxiety	**Longitudinal study:** 18-month follow-up (14–16 years)Individuals that scored higher in Hopelessness and Impulsivity were more likely to report engaging in BD.Elevated levels of Depression symptoms predicted faster rates of increase in alcohol use. High Anxiety sensitivity and Anxiety symptoms showed a faster rate of increase in alcohol use compared with only high Anxiety sensitivity without Anxiety symptoms. Adolescents with elevated levels of Impulsivity and heavier drinking were less likely to a normative decline in Depression symptoms.

Martin et al. (28)	153 women university students 19.72 years.	≥4 drinks in a time during the past two weeks	Ten-Items Personality Inventory for assessed Big Five traits	Openness was the only personality trait related to BD. Women with higher levels of Openness who engaged in extreme exercise, fasting, or purging were more at risk for heavy and problematic alcohol use.

Motos et al. (29)	213 university students binge drinkers (121 women) 18.20 years.	≥40/60 g alcohol for women/men in 2/3 h the past 6 months	Reduced Neuroticism-Extraversion-Openness (NEO) Five Factor Inventory; Behavioral Inhibition System (BISys)	The influence of personality was quite limited.Binge drinkers presented especially high levels of Neuroticism and Extraversion.Impulsivity and Conscientiousness, along with age, explained most of the weekly consumption behavior among men. Only Impulsivity and Neuroticism contributed to explain the consequences of consumption.

Mushquash et al. (30)	317 school students (168 girls), Canadian aboriginal 16.00 years.	≥4/5 drinks for women/men on one occasion	Substance Use Risk Profile Scale (SURP)	Sensation seeking and Hopelessness predicted BD.All personality traits predicted alcohol-related problems.

Pilatti et al. (31)	298 women university students 18.27 years.	≥4 drinks on one occasion last three months	BigFive Questionnaire for Children (BFQ-C); Sensation Seeking Scale (SSS, form V)	Regular drinkers with BD and moderate drinkers had higher scores in Extroversion and alcohol expectancies for social facilitation than abstainers.Regular drinkers with BD exhibited, compared to moderated drinkers, higher scores in Extroversion, Experience seeking, Disinhibition, and alcohol expectancies for social facilitation.

Rush et al. (32)	208 university students (118 women) 56 binge drinkers 18.54 years.	≥4/5 drinks for women/men in a row across at least one sitting in the past month	Neuroticism-Extraversion-Openness (NEO) Five Factor Inventory	Binge drinkers did not differ from non-bingers in the NEO five dimensions when compared with college norms. The comorbidity between binge eating and drinking was related to higher levels of Neuroticism and a tendency to lower Conscientiousness (uncontrolled style of impulse control).

Scaife and Duka (33)	60 young (30 women) moderate and heavy social drinkers 20.6 years.	Score calculate by average drinks per hour, number of time being drunk in the previous 6 months and percentage of time getting drunk when drinking	Revised Neuroticism-Extraversion-Openness (NEO) Five Factor Inventory; Spielberger State-Trait Anxiety Inventory (STAI)	Binge drinkers rated lower in Openness than non-binge drinkers (only approached significance).Anxiety ratings did not show any differences with regard to BD.

Shin et al. (34)	190 young (115 women) from community sample 18–25 years.	≥4/5 drinks for women/men in a row at least 2–3 days per month in the past year	UPPS Impulsive Behavior Scale	Negative urgency and Sensation seeking were positively associated with BD and alcohol use disorders during emerging adulthood.

Shin et al. (35)	268 young (139 women) from community sample 21.9 years.	≥4/5 drinks for women/men in a row at least 2–3 days per month in the past year	UPPS Impulsive Behavior Scale	Negative urgency played a significant role in BD, as well as peer use and parental alcoholism.

Whelan et al. (5)	692 adolescents (312 girls) 14.56 years. at the inclusion	A minimum of three life time episodes leading to drunkenness	Novelty-seeking scale of Temperament and Character Inventory-Revised (TCI-R); Neuroticism-Extraversion-Openness (NEO) Five Factor Inventory; Substance Use Risk Profile Scale (SURP)	**Longitudinal study:** 2-year follow-up (14–16 years).Higher Novelty-seeking (Disorderliness and Extravagance) and lower Conscientiousness characterized both current and future binge drinkers.Agreeableness and Impulsivity classified current but not future binge drinkers.Anxiety sensitivity (SURP) predicted only future BD.

Winograd et al. (36)	988 university students drinkers (494 women) 18.2 years.	≥4/5 drinks for women/men in within a 2 h period	Goldberg’s International Personality Item Pool (IPIP), sober and drunk assessed	Perceived drunken personality associated with less Conscientiousness, Openness, Agreeableness and Neuroticism, and more Extraversion. Women reported larger decreases in Conscientiousness, Openness, and Neuroticism than men. Men reported larger decreases in Agreeableness (more aggressive when drunk).Binge drinkers reported increases in Extraversion (in contrast to decreases in non-binge drinkers), and greater decreases in Neuroticism (more anxiolytic effects) and Agreeableness.

Winograd et al. (37)	374 university students (187 drinking buddies; 202 women) 18.4 years.	≥5 drinks at a single sitting 1 or more times per month	Goldberg’s International Personality Item Pool (IPIP), sober and drunk assessed	Extraversion was positively associated with more frequency of BD and more negative consequences, whereas Conscientiousness was associated with less BD and fewer consequences (sober and drunk).Under intoxication, drinkers reported lower levels of Agreeableness (impaired control of aggressiveness and low empathy), Conscientiousness (lower self-control) and Openness/Intellect, and higher Extraversion (more sociability) and Emotional Stability (more stress-dampening and anxiolytic effects).

Winograd et al. (38)	374 university students (187 drinking buddies; 202 women) 18.4 years.	≥5 drinks at a single sitting in the past 30 days	Goldberg’s International Personality Item Pool (IPIP), sober and drunk assessed	Personality drunk types:(1) Intoxication-related decreases in Conscientiousness and Openness below average. (2) High in Agreeableness when sober and decreasing less than average in Conscientiousness and Openness, and increasing more than average in Extraversion when drunk. (3) Intoxication related to larger decreases in Conscientiousness and Openness and smaller increases in Extraversion. (4) Low in Extraversion when sober and increasing more than average in Extraversion and decreasing more than average in Conscientiousness when drunk.

Zhang et al. (39)	3,110 young adults (1,648 women) at the final recording 30.9 years.	≥5 drinks in a row during the past 12 months	Mini International Personality Item Pool (Mini-IPIP) of the Big Five Personality factors	**Longitudinal study:** 15-year follow-up, participants in grades 9–12 (USA) at inclusion.Risk profiles of personality for frequent BD:(1) Reserved (high Conscientiousness and low Extraversion, Openness and Agreeableness). Those with higher rates of frequent BD may be at risk for type I alcoholism. (2) Resilient (high Extroversion, Openness and especially Agreeableness). Those with a greater tendency toward social contacts may be more vulnerable to social drinking circumstances.

*UPPS Impulsivity: Urgency, Lack of premeditation, Lack of perseverance, and Sensation seeking. UPPS-P considers two facets of Urgency: Positive and Negative*.

### Impulsivity and Sensation Seeking

The two most studied personality traits for BD are Impulsivity and Sensation seeking. Impulsivity is a multidimensional construct associated with poor planning skills, difficulty maintaining attention, and risk-taking behavior. Sensation seeking is defined as the general need for adventure and excitement, the preference for unforeseeable situations and friends, and the willingness to take risks simply for the experience of living them. Many studies have observed higher scores in binge drinkers in both Impulsivity (11, 12, 26, 27, 29) and Sensation seeking (12, 16, 22, 25, 26, 30, 31, 34), when compared with non-binge drinkers. Both traits are considered risk factors for lifetime, whose joint presence has been labeled as “disinhibited personality” (18), although they are especially present in adolescence, characterized by increased impulsive decision making and behavior (40). Similarly, the scores of Impulsivity and Sensation seeking are related to the number of drinks consumed per episode (14, 20, 23) and the frequency of BD (17, 18, 23).

The existing data have been obtained independently of the personality model or the measurement instrument used, either by conceptualizing Impulsivity and Sensation seeking as independent but related features or considering Sensation seeking as a facet of impulsivity. In this second case, the meta-analysis of Stautz and Cooper (40) about the Impulsivity facets as risk factors for problematic alcohol use in adolescence, including BD, were in this order: Sensation seeking, Lack of premeditation, Negative urgency, and Lack of perseverance. These are the dimensions evaluated by the UPPS Impulsive Behavior Scale, frequently used in this field of study. The Negative urgency or tendency to act rashly when experiencing negative emotions is related to BD (34, 35) and is also the only facet related to its severity (16) and to alcohol use disorders as well (34). According to this, BD has been conceptualized as a maladaptive short-term coping strategy devoted to relieving negative affective states (16), which is congruent with the expectations of tension reduction with alcohol that present the binge drinkers, especially in men (14). In the same way, the consideration of facets from Sensation seeking (Thrill and adventure, Experience seeking, Disinhibition, and Boredom susceptibility) indicates that Thrill and adventure and Boredom susceptibility are associated with BD (3). Both facets are externalizing and have psychopathological connections, according to the model of Krueger et al. (41).

A very relevant aspect is that the relationship between BD and Impulsivity and/or Sensation seeking can be modulated by several factors. It should be noted that personality profile of BD could be modulated by sex since the highest levels of Impulsivity and/or Sensation-Seeking come from the men’s scores (11, 12, 29). Moreover, Sensation seeking is the strongest predictor of personality for discriminating binge drinkers from non-drinkers and moderate drinkers in men (22). The expectancies of consumption are mediating in the relationships between the personality traits and BD. Thus, binge drinkers with high Impulsivity show positive expectancies (17), whereas in subjects with high Sensation seeking the greater frequency of episodes of BD is modulated by the positive consequences from drinking (23). Recent work by Lannoy et al. (24) points to the existence of three types of binge drinkers according to their facets of Impulsivity and drinking motives: Emotional (higher Sensation seeking and Urgency), Recreational (higher Lack of Premeditation and Perseverance), and Hazardous (moderate to high drinking motives). This proposal represents an advance with possible practical implications in the future.

### The Big Five Personality Model

This personality model considers five dimensions: Extraversion, Neuroticism/Emotional stability, Conscientiousness, Openness (to new experiences)/Intellect, and Agreeableness. Personality data using the Big Five model are inconclusive in cross-sectional studies of BD. High Extraversion is the feature most consistently associated with BD (22, 29, 31), also being related to a higher frequency of BD and more negative consequences (37). In relation to Conscientiousness, which negatively correlated with impulsivity (42), although binge drinkers exhibit usually low scores (5, 19, 32, 37), high values (especially in men) have been also described (29). In this sense, a lower level of self-oriented Perfectionism, which could be considered as a form of hyper Conscientiousness, has also been observed in BD (21). Low Conscientiousness is considered as associated with less prosocial and more health-promoting behaviors (dietary and lifestyles) in general (43). Finally, high Openness has been related to BD in women (28). Some studies have not found relationships between these personality characteristics and BD (32, 33), although they are characterized for including small samples of BD, basically of social drinkers.

The Neuroticism/Emotional stability is the strongest predictor of personality trait that discriminates between binge drinkers and non-drinkers and moderate drinkers in women (22), with low scores in binge drinkers. This could suggest that a higher emotional instability avoids heavy alcohol intake. However, with the Zuckerman personality model (ZKPQ), a higher Neuroticism-anxiety has been observed in binge drinkers, although this is a consequence of the results from women (12). High levels of Neuroticism also explain the negative consequences of alcohol consumption in both sexes (29). The review by De Wever and Quaglino (44) suggests the need to study further the involvement of affective factors (anxiety and depression), which may be premorbid and appear or are aggravated by the consumption. Neuroticism is precisely the most important personality dimension related to many forms of psychopathology, including anxiety, depression, and substance use disorders (12).

Other traits of interest studied are the type-D personality and the Boredom proneness. The first is characterized by a high tendency toward experiencing negative emotions and inhibiting the expression of emotions and behaviors in social situations. Boredom proneness is associated with undesirable emotional states such as depression, hopelessness, loneliness, amotivational orientation and is negatively related to life satisfaction and autonomy orientation. Both are considered risk variables for mental health, since type-D personality predicts the amount of alcohol consumed (8) and Boredom proneness influences the social expectancies (15) of BD.

### Substance Use Risk Profile Scale (SURP)

In the area of risk for substance consumption, including alcohol, the SURP scale has been developed, which evaluates four dimensions: Anxiety sensitivity, Sensation seeking, Impulsivity, and Hopelessness (a lower order factor of Neuroticism). To a lesser or greater extent, all of these dimensions appear to be implicated as risk factors in BD. In several studies using the SURP, binge drinkers scored higher in Sensation seeking, Impulsivity and Hopelessness than non-bingers (5, 27, 30), and all the personality traits were related to alcohol problems (30). This scale, with very adequate psychometric properties, is the one selected to assess personality in the “Preventure” prevention program, which will be discussed later.

### Changes and Evolution of the Personality Traits Related to BD

In longitudinal studies, Impulsivity and Sensation seeking are prognostic factors for the maintenance and intensification of the BD pattern (5, 13) and alcohol/drug-related problems and other disorders (18, 27). This is observed independently of the personality instrument of measurement. Ashenhurst et al. (13) proposed a deviant pattern of personality maturation without a reduction in both Impulsivity and Sensation seeking as age increases in young adults who developed an increasing trajectory of BD. Anxiety sensitivity also predicts future BD (5). Faster rates of increase in alcohol use have been related to high Anxiety sensitivity and coexisting anxiety symptoms (27).

Zhang et al. (39) have proposed several alcohol consumption trajectories, based on a cohort followed for 15 years, which can give meaning to the heterogeneity of existing results with the Big Five model. These authors suggest two risk profiles, the “Resilient” one, more vulnerable to social pressure for drinking, and the “Reserved” one, with higher risk for alcoholism. The first is characterized by high Agreeableness, Extroversion, and Openness, whereas the second is defined by high Conscientiousness and low Extraversion, Openness, and Agreeableness. High Extraversion also appeared related to BD in other longitudinal study (5).

In connection with the consumption expectations, it is interesting to examine the effects on the perceived personality related to intoxication as compared with the sober state. Using the Big Five personality model, it has been observed that binge drinkers report increases in Extraversion, and greater decreases in Neuroticism (anxiolytic effects) and Agreeableness (more aggressive) than non-binge drinkers, a pattern modulated by sex (36, 37). Four different drunk types have been noted (38), whose consideration in the future may complement the explanatory model of BD (Table 1).

### Interventions Considering Personality Traits

There is no doubt that investing time and resources in promoting health at an early age, prior to the onset of consumption, has positive repercussions, including minimizing the pattern of BD. The alcohol selective prevention program “Preventure,” a brief personality-targeted intervention for youth, is an outstanding example of this strategy (45). This program covers three main components: psychoeducational, motivational interviewing, and cognitive behavioral. The intervention has been particularly effective in preventing the growth of BD in early adolescents of both sexes with high Sensation Seeking and Impulsivity and in girls with higher Anxiety sensitivity. This has been evidenced over 36-month follow-up in Australia (46), at a 24-month postintervention in England (47), and at a 12-month follow-up in the Netherlands (45) to mention only studies with longer follow-up periods.

Although our review is focused on personality, an overall explanatory model of BD must also incorporate attitudes, motives, expectancies, or metacognitions referring to consumption, since these are mediating variables in the relationships between personality and BD (17, 44), in addition to participating in the prediction of alcohol-related problems (23, 30). Binge drinkers, regardless of their personality characteristics, exhibit higher alcohol expectancies for social facilitation (31) and positive metacognitions (19) than regular moderate drinkers and abstainers. This is especially important in selective prevention, in which the restructuring of dysfunctional metacognitions (e.g., drinking alcohol to avoid negative judgments from others) may help in the control of drinking, while the establishment of adaptive emotional regulation strategies (16, 24) may increase the success of the interventions. As a harm reduction strategy, moreover, educating in protective patterns of drinking is effective in reducing the BD frequency in individuals with high Impulsivity and Sensation seeking (20).

Prevention should be initiated at an early school age and not limited to specific actions, since the general objective should be to promote the empowerment and integral health of young people. The inclusion of multiple elements to promote protective factors seems to be the best strategy to revert to healthier habits and a better quality of life in the short and long term. From this perspective, and for a greater success of these approaches, it is necessary to consider the personality characteristics that represent a vulnerability factor for the initiation and maintenance of BD.

## Limitations and Future Directions

There is great heterogeneity in the scales used for personality assessment, based on various theoretical models, which makes it difficult to compare the results of different studies. An effort is required to agree on a measurement instrument that integrates those dimensions or facets that represent the main risk factors in BD. We consider that the use of the SURP is very appropriate. Moreover, when it is complemented with the Big Five dimensions of Conscientiousness (for its relevance in health habits) and Extraversion, it could improve the information collected on personality. Furthermore, only a minority of articles has compared the scores obtained with normative data from their corresponding countries, or in the cases where these do not exist, making some sort of conversion to normative scores (z, T,…). That is, finding higher or lower scores of a certain dimension in BD with respect to another group does not always imply that these are values outside the normal population range.

The epidemiological characteristics of the samples, especially sex, age, and race, are rarely analyzed as factors of interaction with the personality traits associated with BD. Most studies collect this information merely as descriptive of the sample, analyze it independently, or only consider it as a control. It is essential to develop future works that explore the modulating effect of epidemiological variables on representative samples, since the studies that have done so have pointed out that the data are not generalizable to the entire population.

It is also required to consider and control for other variables that are known to influence the appearance and maintenance of BD when they are not the objective of the study, highlighting the presence of psychiatric symptomatology or mental disorders, stressful life events, and circadian rhythmicity. In relation to the latter, an adequate sleep (48) and a morning typology (49) are protective factors for heavy drinking and for extreme personality traits.

The development of longitudinal studies, a minority to date, is the only way to elucidate the specific weight of the personality traits in the initiation and maintenance of BD and/or relate problems, as well as the impact of BD practice in personality. At the same time, this would allow us to define the age with the greatest vulnerability and the best time for the implementation of prevention programs.

For an integral and explanatory perspective of BD, studies should integrate also biological and neurocognitive evaluations. BD is not a unitary phenomenon but consists of a combination of history, personality, and brain domains (5), and this is how it should be examined. Only this approach will help to delineate subgroups of risk for BD and to interpret different trajectories and consequences of its practice in the short, medium, and long term.

Finally, multicenter and multicountry studies will allow us to explore whether there are sociocultural differences in BD, and whether these require specific adaptations in both preventive and treatment approaches. The “Preventure” program, for example, has only been carried out in Anglo-Saxon countries and its development in a Mediterranean or Latin American country may lead to different effectiveness and may require some methodological adjustment.

## Author Contributions

AA and JFN collected the materials and resources needed for this review and wrote this article. DAF provided suggestions and revised each draft of the manuscript.

## Conflict of Interest Statement

The authors declare that the research was conducted in the absence of any commercial or financial relationships that could be construed as a potential conflict of interest.
